# Agreement and Precision of Wide and Cube Scan Measurements between Swept-source and Spectral-domain OCT in Normal and Glaucoma Eyes

**DOI:** 10.21203/rs.3.rs-3002468/v1

**Published:** 2023-06-06

**Authors:** Huiyuan Hou, Nevin W. EI-Nimri, Mary K. Durbin, Juan D. Arias, Sasan Moghimi, Robert N. Weinreb

**Affiliations:** Topcon Healthcare; Topcon Healthcare; Topcon Healthcare; Topcon Healthcare; Shiley Eye Institute, University of California; Shiley Eye Institute, University of California

## Abstract

This study aimed to evaluate agreement of Wide scan measurements from swept-source optical coherence tomography(SS-OCT) Triton and spectral-domain OCT(SD-OCT) Maestro in normal/glaucoma eyes, and to assess the precision of measurements from Wide and Cube scans of both devices. Three Triton and three Maestro operator/device configurations were created by pairing three operators, with study eye and testing order randomized. Three scans were captured for Wide (12mm×9mm), Macular Cube (7mmx7mm–Triton; 6mmx6mm-Maestro), and Optic Disc Cube (6mmx6mm) scans for 25 normal eyes and 25 glaucoma eyes. Thickness of circumpapillary retinal nerve fiber layer(cpRNFL), ganglion cell layer+inner plexiform layer(GCL+), and ganglion cell complex(GCL++) was obtained from each scan. A two-way random effect analysis of variance model was used to estimate the repeatability and reproducibility; agreement was evaluated by Bland-Altman analysis and Deming regression. Precision limit estimates were low: <5μm for macular and <10μm for optic disc parameters. Precision for Wide and Cube scans of both devices were comparablein both groups. Excellent agreement between the two devices was found for Wide scans, with the mean difference<3μm across all measurements (cpRNFL<3μm, GCL+<2μm, GCL++<1μm), indicating interoperability. A single Wide scan covering the peripapillary and macular regions may be useful for glaucoma management.

## Introduction

Primary open angle glaucoma (POAG) is characterized by progressive loss of retinal ganglion cells (RGCs) and their axons, and accompanying damage to the visual field (VF).^1^ For most clinicians, management of glaucoma usually requires both a functional test, in particular, a VF obtained by automated perimetry, and a structural test, most commonly with optical coherence tomography (OCT) imaging.^2^ In addition to the useful OCT cross-sectional B scans, which allow for visualization of retinal structures, thickness measurements facilitate quantitative evaluation of the neural retina affected by glaucomatous damage. Thus, OCT is an irreplaceable imaging technology for glaucoma diagnosis and management.

Early detection and close monitoring of glaucomatous damage are important to avoid irreversible vision loss. Characteristic excavation and narrowing of the neuroretina rim of the optic disc as a result of nerve degeneration is the most well-known glaucomatous manifestation. Several studies have also suggested that glaucomatous damage to the macula, where more than 30% of the RGCs in the eye reside,^3^ is common and can occur early in the disease.^4,5^ In addition, damage to the macular RGCs and peripapillary retinal nerve fiber layer (RNFL) likely follow certain patterns that are closely related with each other. Although a number of studies have found that measures of macular RGC and peripapillary RNFL thickness have similar sensitivity and specificity,^4^ it is not expected for these measures to provide equivalent information. Therefore, clinicians need to be aware that measurements of both the peripapillary and macular regions are considered an indispensable part of the comprehensive evaluation of glaucomatous damage.^4^

Clinically available OCTs have various scan protocols for macular and optic disc measures. Typically, the macula and peripapillary regions are scanned independently to obtain the respective measures. However, it is useful to have methods for combining the information from both macular RGC and peripapillary RNFL measures in a single scan to aid clinical decision-making by recognizing pattens of glaucomatous damage.^4^ Current generation OCTs allow wide field visualization of the retina with a scan area that encompasses both the macula and optic disc, making the clinical workflow for the technology more efficient and facilitating the investigation of macular and peripapillary OCT measurements simultaneously.^6–8^ Studies have shown that a wide field scan has glaucoma-discriminating ability comparable to a combination of more dense (macula and peripapillary) Cube scans.^6–8^ While the precision of macula and optic disc Cube scans has been well investigated,^9–12^ information on the repeatability and reproducibility of Wide scan measurements is limited. Moreover, even though both Swept-Source OCT (SS-OCT) and Spectral-Domain OCT (SD-OCT) technologies offer a Wide scan mode, no prior study has compared Wide scan measurements between these two devices. Considering that SD-OCT and SS-OCT devices are both abundantly accessible in eye care clinics and research environments, interoperability of their data would enhance clinical care and research.^13^

The purpose of this study was to evaluate the agreement of Wide scan measurements between Triton SS-OCT and Maestro SD-OCT and to assess the repeatability and reproducibility of measurements from the Wide scan and the Macula/Optic Disc Cube scans of the two devices in normal and glaucoma eyes, and to further evaluate the interoperability of these two technologies.

## Methods

This was a prospective study. Subjects signed an informed consent form and fulfilled all inclusion and exclusion criteria. The IntegReview Institutional Review Board (3815 S. Capital of Texas Hwy, Suite 320, Austin, TX 78704) approved the study protocol, and the methodology adhered to the tenets of the Declaration of Helsinki for research involving human subjects and to the Health Insurance Portability and Accountability Act.

### Participants

Subjects underwent an ocular examination to determine eligibility for study enrollment. Assessments included best-corrected visual acuity (BCVA), refraction, slit lamp biomicroscopy, ophthalmoscopy, intraocular pressure (IOP), and VF (standard automated perimetry, Humphrey Field Analyzer; 24 − 2 Swedish interactive threshold algorithm - standard; Carl Zeiss Meditec, Inc., Dublin, California).

Overall inclusion criteria for subjects in this study were 18 years of age or older on the date of informed consent, ability to understand the written informed consent, and willingness to participate as evidenced by signing the informed consent. All eligible subjects had a bilateral BCVA of 20/40 or better. Subjects were excluded if they were unable to tolerate ophthalmic imaging, had ocular media that precluded acceptable OCT images, history of leukemia, dementia or multiple sclerosis, or concomitant use of hydroxychloroquine or chloroquine. Subjects were excluded from the Glaucoma group if their 24 − 2 VF result was unreliable (defined as fixation losses > 20%, false positives > 33%, or false negatives > 33%).

Subjects in the Normal group were defined as presenting with normal eyes bilaterally (non-visually impairing cataract was acceptable). Subjects had IOP ≤ 21 mmHg in each eye. Clinically defined normal subjects that had VF defects consistent with glaucomatous optic nerve damage based on at least one of the following findings or narrow angles in either eye were excluded from the Normal group: a) On pattern deviation (PD), there exists a cluster of 3 or more points in an expected location of the VF depressed below the 5% level, at least 1 of which is depressed below the 1% level; b) Glaucoma hemi-field test “outside normal limits”.

Subjects included in the Glaucoma group were defined as those with VF defects as described above consistent with glaucomatous optic nerve damage and having glaucomatous optic nerve damage as clinically evidenced by any of the following optic disc or RNFL structural abnormalities: a) Diffuse thinning, focal narrowing, or notching of the optic disc rim especially at the inferior or superior poles with or without disc hemorrhage; b) Localized abnormalities of the peripapillary RNFL, especially at the inferior or superior poles; c) Optic disc neural rim asymmetry of the two eyes consistent with loss of neural tissue.

Subjects were excluded from the Glaucoma group if they presented with presence of any ocular pathology except glaucoma in the study eye (non-visually impairing cataract was acceptable).

The study eye and the testing order of the operator/device configuration were randomized for each subject. If only one eye of a subject in the Glaucoma group had pathology and met the eligibility criteria, the eligible eye was the study eye. For eyes in the Normal group, both eyes were required to have met all normal eligibility criteria prior to study eye randomization.

### Optical Coherence Tomography Scans

**Figure 1:**
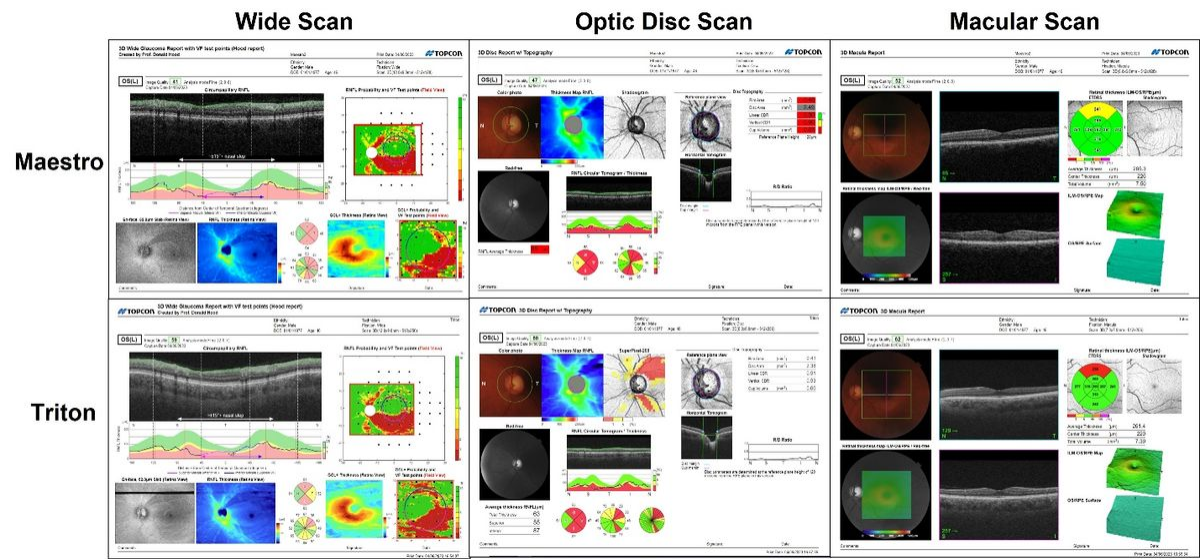
Representative OCT reports of Wide scan and Cube scans from one glaucoma eye (24–2 visual field mean deviation −3.56dB). The top row shows the reports of (left to right) Wide scan (12mm×9mm), Optic Disc Cube scan (6mm×6mm), and Macular Cube scan (6mm×6mm) from the Maestro; and the bottom row shows the reports of (left to right) Wide scan (12mm×9mm), Optic Disc Cube scan (6mm×6mm), and Macular Cube scan (7mm×7mm) from the Triton.

This study included three SS-OCT devices (DRI OCT Triton, Topcon Inc, Tokyo, Japan) and three SD-OCT devices (3D OCT-1 Maestro, Topcon Inc, Tokyo, Japan). Three operators used these study devices to acquire the scans. Each operator was paired with one specific DRI OCT Triton and one specific 3D OCT-1 Maestro to create three distinct operator/device configurations. The OCT imaging was conducted during a single session. The scan types included for the Triton were Wide scan (12mm×9mm), Optic Disc Cube (6mm×6mm), and Macular Cube (7mm ×7mm) scans. The scan types included for the Maestro were Wide scan (12mm×9mm), Optic Disc Cube (6mm×6mm), and Macular Cube (6mm ×6mm) scans. Figure 1 shows representative OCT reports of these scan types from Maestro and Triton. Circumpapillary RNFL (cpRNFL) thickness measurements were derived from the Wide and Optic Disc Cube scans. Macula ganglion cell layer (GCL) and inner plexiform layer (IPL) (mGCIPL/mGCL+) thickness and macula ganglion cell complex (mGCC/mGCL++) thickness were derived from the Wide and Macular Cube scans. For each scan type, at least 3 scans per eye for each operator/device configuration were taken.

All scans of each study subject were evaluated by two independent masked imaging experts in a randomized fashion for image quality acceptance. The graders were blinded to operator, device, subject ID, subject data, and each other’s grading. Individual B scan evaluation was conducted on the central horizontal B scan from the first of the three volume scans captured from each scan type (Wide, Macular Cube, Optic Disc Cube). Any scan deemed unacceptable was not included in the data analysis.

### Statistical Analysis

In general, descriptive statistics (n, mean, standard deviation (SD), and median) were used to summarize continuous variables. Frequencies and percentages were used to summarize categorical variables.

The precision analysis was conducted utilizing all acceptable scans for both the Triton SS-OCT and Maestro SD-OCT for all scan types. A two-way random effect analysis of variance (ANOVA) model was used to estimate the repeatability and reproducibility of each scan parameter by group and study device (Triton and Maestro). This ANOVA model included the operator/device, eye, and interaction between operator/device and eye as the variance components. The repeatability and reproducibility limits and coefficient of variation in percentage (CV%) were produced for each scan parameter by group and device. Specifically, repeatability SD = square root of the residual variance; reproducibility SD = square root of the sum of the operator/device variance, the interaction variance, and the residual variance; repeatability limit = 2.8 × repeatability SD; reproducibility limit = 2.8 × reproducibility SD; repeatability CV% = (repeatability SD)/intercept × 100%; reproducibility CV% = (reproducibility SD)/intercept × 100%.

Analysis of agreement included the first acceptable scan from each scan type (Wide scan, Macular Cube scan, and Optic Disc Cube scan) from the Triton and Maestro. Agreement between the two devices was evaluated using Bland-Altman plots to calculate the mean difference and the limits of agreement (LOA), and Deming regression to calculate slope and intercept, for each scan type in each group.

Statistical analyses were performed using statistical software SAS 9.3 or later (SAS Institute, Cary, North Carolina). P values less than 0.05 were considered statistically significant.

## Results

### Clinical demographics

25 Normal subjects (25 eyes) and 25 Glaucoma subjects (25 eyes) were included. Demographic and ocular parameters of the study subjects are summarized in Table 1. The mean overall age was 59.3 ± 16.8 years, with the Glaucoma group being older. The study population was almost equally distributed between genders (52% male and 48% female). Most study eyes (78%) had a BCVA of 20/20 or better. For eyes in the Glaucoma group, the mean 24 − 2 mean deviation (MD) was − 5.30 ± 6.77dB and the mean pattern standard deviation (PSD) was 4.70 ± 2.87dB.

### Repeatability and reproducibility of measurements from Wide scan and Cube scan

Precision including repeatability and reproducibility of measurements from Wide and Cube scans was evaluated and compared. Overall, the precision estimates (reproducibility limit, reproducibility CV%, repeatability limit and repeatability CV%) were low, indicating high precision for all measurements (cpRNFL, GCL+, and GCL + + thickness). Precisions of the Wide and Cube scans were mostly similar in each device.

Table 2 summarizes the repeatability and reproducibility estimates of GCL + thickness measurements in Normal and Glaucoma groups. In the Normal group, the CV% for repeatability and reproducibility of both Triton and Maestro OCT devices ranged between 0 and 1% with the exception of the reproducibility CV% for inferior thickness from the Maestro Wide scan, which was 1.1%. The CV%s were generally higher in the Glaucoma group with a range of repeatability CV% between 0.6 to 1.7% for the Triton Wide scan and 0.5 to 1.4% for the Triton Macular Cube scan. In the Normal group, the limit ratios between Wide scan and Macular Cube scan for both OCT devices mostly ranged from 0.7 to 1.3, indicating comparable precision between scan types. In the Glaucoma group, the corresponding limit ratios for repeatability and reproducibility on the Triton ranged from 0.9 to1.8 (average GCL + thickness, 1.3) and 0.9 to1.6 (average GCL + thickness, 1.1), respectively, compared to 1.2 to 3.0 (average GCL + thickness, 2.2) and 1.3 to 2.7 (average GCL + thickness, 2.1), respectively for the Maestro.

Supplementary Table 1 summarizes the repeatability and reproducibility estimates of GCL + + thickness measurements in the Normal and Glaucoma groups. GCL + + thickness measurements from both scan types in the two devices showed excellent precision in the Normal and Glaucoma groups with CV% of repeatability and reproducibility within 1%, with the exception of CV% of reproducibility on the Maestro for the Superior Nasal region in the Glaucoma group, which was 1.1%. The repeatability limit of the Wide scan ranged from 1.1μm to 2.1μm for the Triton and from 1.2μm to 2.1μm for the Maestro. The reproducibility limit of the Wide scan ranged from 1.6μm to 2.4μm for the Triton and from 1.4μm to 2.9μm for the Maestro in both groups. All the limit ratios (between Wide scans and Macular Cube scans) in the Normal group were around + 1 (maximum 1.3) for both the Triton and Maestro OCT devices. In the Glaucoma group, the limit ratios for repeatability and reproducibility on the Triton ranged from 0.9 to 1.1 (average GCL + + thickness, 1.1) and 0.9 to 1.1 (average GCL + + thickness, 1.0), respectively, and 0.7 to 1.4 (average GCL + + thickness, 1.1) and 0.7 to 1.2 (average GCL + + thickness, 0.9) on the Maestro, indicating that the GCL + + thickness measurements from the Wide scan and Macular Cube scan had similar precision in both devices.

Table 3 shows the repeatability and reproducibility estimates of RNFL thickness measurements in the Normal and Glaucoma groups. Overall, precision of RNFL thickness was inferior to that of macular measurements in both the Normal and Glaucoma groups. The CV% for repeatability and reproducibility of the Triton Wide scan measures in glaucoma eyes ranged between 1.4 to 2.9% and 1.6 to 3.4% respectively, similar with the Optic Disc Cube scan (1.3 to 2.9% and 1.7 to 3.2%, respectively). All the limit estimates for the Triton were < 10 μm in both groups; repeatability and reproducibility limits of Wide scan measurements for glaucoma eyes ranged between 3.0 to 6.5μm and 3.4 to 7.2μm, respectively). In comparison, the Maestro showed slightly higher limit estimates (maximum 11.8μm). All limit ratios for Wide scan and Optic Disc Cube scan were around + 1, indicating minor difference of the limit between the two scan types. In the Glaucoma group, the limit ratios of repeatability and reproducibility for the Triton ranged between 0.9 to 1.3 (average RNFL thickness, 1.1) and 0.9 to 1.2 (average RNFL thickness, 1.0), respectively, and 0.9 to 1.4 (average RNFL thickness, 1.2) and 0.9 to 1.4 (average RNFL thickness, 1.3), respectively, for the Maestro, indicating that the RNFL thickness measurements from the Wide scan and Optic Disc Cube scan had similar precision.

### Agreement of Wide scan measurements between SS-OCT and SD-OCT

Assessment of Wide scan macular and peripapillary RNFL thickness agreement showed that the measurement differences between the Triton and Maestro were small across all parameters. Supplementary Tables 2 and 3 summarized agreements of GCL+, GCL++, and cpRNFL thickness measurements from the Wide scan between Triton and Maestro. The mean differences of GCL + thickness and GCL + + thickness between the two devices were < 2μm and < 1μm, respectively, in both groups. For GCL + thickness measurements, Triton had slightly lower measurements than Maestro in both groups. GCL + + thickness measurements were slightly higher for Triton in the Glaucoma group, but more comparable in the Normal Group. From Supplementary Table 3, RNFL thickness measurements were slightly higher for Triton in the Glaucoma group (mean difference between the two devices < 3μm), and slightly lower in the Normal group except for nasal sector. All the differences were very minimal and not statistically significant.

**Figure 2:**
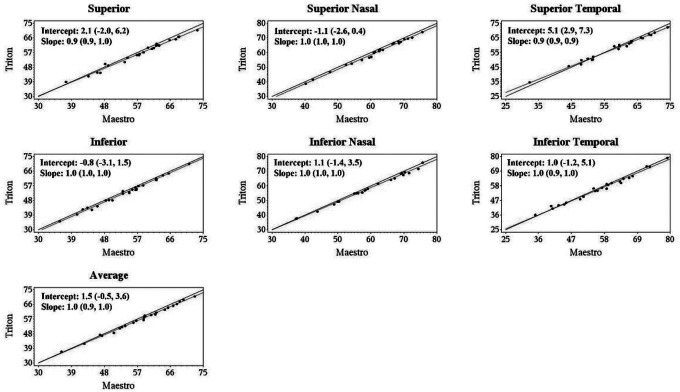
Deming regression plot of thickness of ganglion cell layer and inner plexiform layer (GCIPL/GCL+) from the Wide scan of Triton and Maestro in glaucoma eyes. Intercepts and slopes of the fitted linear model are shown as mean (95% confidence interval) and indicate excellent agreement of the Wide scan measurements between Triton and Maestro.

**Figure 3:**
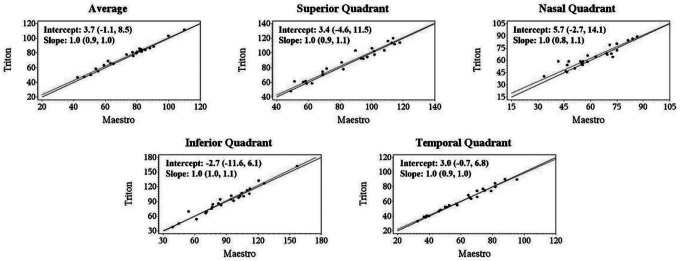
Deming regression plot of thickness measurements of circumpapillary retinal nerve fiber layer (cpRNFL) from the Wide scan between Triton and Maestro in glaucoma eyes. Intercepts and slopes, which are shown as mean (95% confidence interval), indicate measurements of the two devices were in excellent agreement.

Deming regression showed that all the slopes for the Triton and Maestro were close to + 1, and most of the 95% confidence intervals (CIs) for the intercept and slope contained 0 and 1, respectively, that is, the intercepts did not significantly differ from 0 and slopes did not differ significantly from 1, indicating excellent agreement of the Wide scan measurements between Triton and Maestro. Representative Deming regression plots are shown in Figs. 2, Fig. 3, and Supplementary Fig. 1, which illustrate agreement of GCL+, cpRNFL, and GCL + + thickness measurements from the Wide scan between Triton and Maestro in the Glaucoma group, respectively.

## Discussion

This study demonstrated that Wide scan measurements from the Triton SS-OCT and Maestro SD-OCT have excellent agreement in both normal and glaucoma eyes. In addition, the repeatability and reproducibility of cpRNFL, macular GCIPL, and macular GCC thickness measurements from the Wide and the Macular/ Optic Disc Cube scans were similar for both the Triton and the Maestro in normal and glaucoma eyes.

Measuring structural changes is essential for diagnosing and monitoring glaucoma,^1^ and OCT is a well-established method to objectively assess structural changes in eyes with glaucoma.^14^ Before the introduction of a Wide scan that simultaneously captures peripapillary and macular anatomical structures in one scan, separate Macular and Optic Disc Cube scans were required to quantitively assess macular RGCs and RNFL respectively. However, in clinical practice, often only one OCT scan, typically the Optic Disc Cube scan, is captured due to time limitations.^7^ Rather than acquiring data with both Macular and Optic Disc Cube scans, the Wide scan and its incorporated automated segmentation software makes it possible to analyze the thickness of peripapillary RNFL and various macular retinal layers simultaneously using data obtained with only a single scan. As found in the current study, the Wide scan and Cube scan measurements are comparable. Moreover, imaging time is reduced with simultaneous imaging of the macula and the peripapillary region relative to the capturing of two scans per eye. This also minimizes the adverse impact on image quality caused by patient fatigue, motion, and alignment errors. In addition, the paracentral fixation target of the Wide scan reduces the fixation errors caused during acquisition of the Optic Disc Cube scan which requires nasal fixation.^8,15^ Another strength of the Wide scan is that peripapillary RNFL defects at 11 and 12 o’clock (in the right eye orientation) can be missed in the Macular Cube scans, while they are more likely visualized in a Wide scan including both cpRNFL and macular GCC.^16^ Another study has shown that the thickness map provided for Wide scans detects early structural changes that might not be detected well using peripapillary RNFL or macular GCIPL thickness maps. Furthermore, RNFL defects distant from the optic disc can be more easily visualized with the Wide scan RNFL maps.^17^

The diagnostic power of the Wide scan has been evaluated by several studies. Yang et al.^18^ compared the diagnostic ability (healthy vs glaucoma) of RNFL^18^ and macular GCIPL and macular GCC^19^ thickness values from a SS-OCT Wide scan (DRI-OCT, Topcon) and Macular/Optic Disc Cube scans of a SD-OCT (Spectralis, Heidelberg Engineering and Cirrus HD-OCT, Carl Zeiss Meditec), and reported that RNFL, macular GCIPL, and macular GCC thickness values from the Wide scan measured by SS-OCT had similar diagnostic accuracy to Macular/ Optic Disc Cube scan measurements obtained by SD-OCT. Similarly, another study showed the diagnostic ability of SS-OCT (DRI-OCT-1 Atlantis, Topcon) Wide scan measurements for distinguishing preperimetric and early glaucoma from healthy eyes was similar to Macular/ Optic Disc Cube scan measurements from SD-OCT (Cirrus HD-OCT, Carl Zeiss Meditec).^6^ These previous studies compared diagnostic ability of SS-OCT Wide scan with SD-OCT Cube scan, while Hong et al.^8^ compared glaucoma-discriminating ability of measurements from Wide scans with Macular/ Optic Disc Cube scans of SS-OCT (DRI-OCT-1 Atlantis, Topcon) and reported that they were comparable. Furthermore, Hood et al.^7^ reported that the report based upon a single Wide scan has the information needed to diagnose early glaucoma with excellent sensitivity and specificity. Thus, it has been suggested that the Wide scan could replace Macular/ Optic Disc Cube scans for diagnosing and screening glaucoma.^7,8^

Besides discrimination between normal and glaucoma, monitoring patients with glaucoma to detect progression is the mainstay of glaucoma care, which requires reliable measures with good repeatability and reproducibility.^20^ Studies addressing measurement precision of Wide scans are limited. One study^15^ using SD-OCT (Canon OCT-HS100, Canon Europe) compared repeatability of measurements from a Wide scan (13mm×10mm) and Cube scans (Macular scan 10mm×10mm, Optic Disc scan 6mm×6mm) in healthy eyes. Different from our results, they found a 2–3 times larger repeatability limit of the Wide scan compared with the Cube scans. The authors attributed their result partially to the scan density in the Wide scan, which is 4.4 times less than for the individual Cube scans. By contrast, the Wide scan and Cube scan in the current study are closer in scan density. It has also been reported previously that the scan direction affects precision, where horizontal scans have better repeatability than vertical scans.^21^ The Wide scan in the prior study employed vertical B-scans, while the Optic Disc and Macular Cube scans were captured horizontally and vertically, respectively,^15^ thereby potentially contributing to the varied repeatability between the Wide and Cube scans. With SS-OCT (DRI-OCT-1 Atlantis, Topcon), another study^8^, found comparably good repeatability of macular GCIPL and macular GCC thickness values from the Wide scan and Cube scans in healthy and glaucoma eyes; the current results are consistent with these earlier ones. The current study expands on that earlier one as it evaluated repeatability and reproducibility in normal and glaucoma eyes. It shows comparable precision of parameters relevant for glaucoma management between the Wide scan and Macular and Optic Disc Cube scans for both the Triton and Maestro OCT devices.

Agreement between SS-OCT and SD-OCT, as well as agreement between the Wide scan and Macular/ Optic Disc Cube scans have been previously studied. Lee SY et al.^22^ evaluated agreement between SS-OCT and SD-OCT Cube scans, and Lee WJ et al.^6^ evaluated agreement between SS-OCT Wide scan and SD-OCT Cube scans, respectively, in normal eyes using the same devices (SS-OCT: DRI-OCT-1 Atlantis, Topcon vs. SD-OCT: Cirrus HD-OCT, Carl Zeiss Meditec); while Yang et al.^19^ evaluated agreement between SS-OCT Wide scan (DRI-OCT, Topcon) and SD-OCT Cube scans (Cirrus HD-OCT, Carl Zeiss Meditec) for healthy and glaucomatous eyes. In all of these studies, the comparison included different manufacturers, algorithms, and measurement positions and grids. In contrast, the Triton and Maestro share segmentation algorithms and measurement locations. That may explain why the differences observed in this study were generally smaller than those in previous studies. Importantly, the differences between Triton and Maestro (mean difference of all measurements < 3 μm) were less than the axial resolution in tissue (Triton axial resolution 8μm, Maestro axial resolution 6 μm^23^), and smaller than the corresponding reproducibility limits. Therefore, these differences are assumed not to be clinically significant. The reported agreement results were expected based on minor differences in axial resolution, software and algorithm, and a minimal difference in the pixel calibration factor between Triton and Maestro. In addition, Hong et al.^8^ found excellent agreement for macular GCIPL, macular GCC and peripapillary RNFL measurements between the SS-OCT (DRI-OCT-1 Atlantis, Topcon) Wide scan and Cube scans for healthy and glaucomatous eyes; Dominguez-Vicent et al.^15^ showed that measurement differences between the Wide and Cube scans for SD-OCT (Canon OCT-HS100, Canon Europe) were mostly lower than the axial resolution of the device for healthy eyes. In summary, these studies suggested that, for glaucoma follow up, consistency of scan type, device, and OCT technology need to be considered. Although the same device and scan type is optimal, this study demonstrates that measurement interchangeability may be expected within certain configurations of scan types and devices, such as the 12mm ×9mm Wide scan of the Triton SS-OCT and the Maestro SD-OCT.

There are several limitations of this study. First, the results of this study were obtained entirely from Caucasian subjects. Although we do not expect ethnicity to directly affect repeatability or reproducibility, additional studies using subjects from different populations would generalize our conclusions. Second, there was a significant difference in age distributions between Normal and Glaucoma groups. The influence of the inter-group age difference on the current study results (from inter-scan type and inter-device analyses) is negligible because all estimates were presented for each single group without inter-group comparison. The range of retinal thickness measurements of normal subjects from this study were highly similar with that in other publications.^24–26^ Moreover, although the retinal thickness measurements decrease with age (total retina thinning 0.53 μm/year; RNFL thinning 0.44 μm/year),^27^ there is no evidence that the rate of age-related thinning varies in different age groups. Therefore, even if test interval is long enough to affect the evaluation of repeatability and reproducibility, which is not applicable for the current study, the effect should be equal between the groups. Nevertheless, one should take the applicable age range into consideration when interpreting the values of retinal thickness of each group. Third, the glaucoma patients included in this study had an average MD of −5.30 dB indicating that most subjects had early to moderate glaucoma. Studies with a wider distribution of glaucoma severity are needed to evaluate the utility of the Wide scan in advanced disease. Fourth, the sample size of the current study is relatively small with 25 eyes in each group. The sample size was determined based on the 95% LOA and the ANOVA model for precision and 21 eyes per group were deemed appropriate. Lastly, although this study suggests a potential role of a Wide scan for glaucoma monitoring, this cross-sectional study was unable to evaluate how well the Wide scan measurements of Triton and Maestro can identify glaucomatous progression. Longitudinal studies are needed to further evaluate the clinical utility of Wide scans in monitoring glaucoma progression.

In conclusion, we have demonstrated high and comparable precision of peripapillary and macula thickness measurements from Wide, Macular Cube, and Optic Disc Cube scans of the Triton SS-OCT and the Maestro SD-OCT in normal and glaucoma eyes. Wide scan measurements of the Triton SS-OCT and Maestro SD-OCT were interchangeable with excellent agreement. These findings show the potential for more simultaneous evaluation of both macular and peripapillary retinal anatomy from a single Wide OCT scan rather than the clinical standard of capturing an Optic Disc Cube scan and a Macular Cube scan for glaucoma diagnosis and management.

## Figures and Tables

**Table 1:** Demographics and Ocular Characteristics of Study Groups

	Normal	Glaucoma
**By subject** (No.)	25	25
Age (years)		
Mean ± SD / Median	42.7 ±14.7 / 40.0	67.6 ±10.4 / 68.0
Age group, no. (%)		
< 65 years	24 (96)	10 (40)
≥ 65 years	1 (4)	15 (60)
Gender (M/F)	14/11	12/13
Race, Caucasian, no. (%)	25 (100)	25 (100)
**By Eye** (No.)	25	25
BCVA, no. (%)		
20/20 or better	24 (96)	15 (60)
20/21 – 20/40	1 (4)	10 (40)
MRSE (D)		
Mean ± SD	−0.70 ±1.61	−1.07 ± 1.83
Axial Length (mm)		
Mean ± SD / Median	23.93 ±1.08 / 23.72	24.81 ±1.28 / 24.86
IOP (mmHg)		
Mean ± SD / Median	15.2 ±3.1 / 16.0	14.4 ±2.8 / 15.0
24 − 2 Visual Field MD (dB)		
Mean ± SD	N/A	−5.30 ± 6.77
24 − 2 Visual Field PSD (dB)		
Mean ± SD	N/A	4.70 ±2.87

Abbreviations: SD, standard deviation; BCVA, best corrected visual acuity; D, diopter; MRSE, manifest refraction spherical equivalent; IOP, intraocular pressure; MD, mean deviation; PSD, pattern standard deviation.

**Table 2:** Repeatability and Reproducibility of Ganglion Cell and Internal Plexiform Layer Thickness Measurements

	Triton 12×9mm^2^ Wide Scan			Triton 7×7mm^2^ Macular Scan			Limit Ratio*	Maestro 12×9mm^2^ Wide Scan			Maestro 6×6mm^2^ Macular Scan			Limit Ratio*
	SD	Limit	CV%	SD	Limit	CV%		SD	Limit	CV%	SD	Limit	CV%	
**Repeatability**														
**Normal Group**														
Average	0.3	0.7	0.4	0.2	0.6	0.3	1.2	0.2	0.6	0.3	0.3	0.7	0.3	0.9
Superior	0.5	1.5	0.8	0.4	1.2	0.6	1.3	0.6	1.8	0.9	0.5	1.5	0.7	1.2
Superior Nasal	0.4	1.1	0.5	0.4	1.1	0.5	1.0	0.5	1.4	0.7	0.5	1.3	0.6	1.1
Superior Temporal	0.5	1.4	0.7	0.6	1.7	0.8	0.8	0.5	1.4	0.7	0.6	1.6	0.8	0.9
Inferior	0.4	1.1	0.6	0.5	1.3	0.7	0.8	0.6	1.6	0.8	0.5	1.4	0.7	1.1
Inferior Nasal	0.4	1.0	0.5	0.5	1.3	0.6	0.8	0.6	1.6	0.8	0.4	1.2	0.6	1.3
Inferior Temporal	0.5	1.4	0.7	0.7	1.8	0.9	0.8	0.5	1.5	0.7	0.5	1.4	0.7	1.1
**Glaucoma Group**														
Average	0.4	1.0	0.6	0.3	0.8	0.5	1.3	0.8	2.2	1.3	0.4	1.0	0.6	2.2
Superior	0.9	2.5	1.6	0.5	1.4	0.9	1.8	1.1	3.2	2.0	0.6	1.7	1.1	1.9
Superior Nasal	0.5	1.3	0.7	0.4	1.2	0.7	1.1	0.7	1.9	1.1	0.4	1.2	0.7	1.6
Superior Temporal	0.8	2.1	1.3	0.8	2.3	1.4	0.9	1.3	3.7	2.3	0.7	2.1	1.2	1.8
Inferior	0.6	1.7	1.1	0.5	1.5	1.0	1.1	1.0	2.7	1.8	0.8	2.3	1.5	1.2
Inferior Nasal	0.6	1.6	1.0	0.6	1.8	1.1	0.9	0.9	2.6	1.6	0.7	2.0	1.2	1.3
Inferior Temporal	0.9	2.6	1.7	0.8	2.3	1.4	1.1	2.1	6.0	3.8	0.7	2.0	1.2	3.0
**Reproducibility**														
**Normal Group**														
Average	0.3	1.0	0.5	0.4	1.1	0.5	0.9	0.3	0.9	0.5	0.4	1.1	0.5	0.8
Superior	0.7	1.9	1.0	0.6	1.7	0.9	1.1	0.7	2.0	1.0	0.6	1.7	0.8	1.2
Superior Nasal	0.5	1.4	0.7	0.5	1.4	0.7	1.0	0.6	1.7	0.8	0.5	1.5	0.7	1.1
Superior Temporal	0.6	1.7	0.8	0.7	2.0	1.0	0.9	0.6	1.7	0.8	0.6	1.7	0.9	1.0
Inferior	0.5	1.3	0.7	0.6	1.7	0.9	0.8	0.8	2.1	1.1	0.7	2.0	1.0	1.1
Inferior Nasal	0.4	1.2	0.6	0.6	1.7	0.8	0.7	0.7	1.9	0.9	0.6	1.6	0.8	1.2
**Repeatability**														
Inferior Temporal	0.6	1.6	0.8	0.8	2.1	1.0	0.8	0.6	1.7	0.8	0.6	1.8	0.8	0.9
**Glaucoma Group**														
Average	0.5	1.3	0.8	0.4	1.2	0.7	1.1	0.9	2.5	1.5	0.4	1.2	0.7	2.1
Superior	1.0	2.8	1.8	0.7	1.8	1.2	1.6	1.3	3.7	2.3	0.7	1.8	1.1	2.1
Superior Nasal	0.5	1.5	0.9	0.5	1.4	0.8	1.1	0.7	2.0	1.2	0.5	1.5	0.9	1.3
Superior Temporal	0.9	2.4	1.5	0.9	2.4	1.4	1.0	1.5	4.1	2.5	0.8	2.2	1.3	1.9
Inferior	0.7	1.8	1.2	0.7	2.0	1.3	0.9	1.1	3.1	2.0	0.9	2.5	1.6	1.9
Inferior Nasal	0.6	1.8	1.1	0.8	2.1	1.3	0.9	0.9	2.6	1.6	0.8	2.2	1.3	1.2
Inferior Temporal	1.1	3.1	2.0	1.0	2.8	1.7	1.1	2.4	6.8	4.3	0.9	2.5	1.5	2.7

* Limit Ratio: limit of Wide scan / limit of Cube scan. Unit of SD and limit is μm. Abbreviations: SD, standard deviation.

**Table 3:** Repeatability and Reproducibility of Retinal Nerve Fiber Layer Thickness Measurements

	Triton 12×9mm^2^ Wide Scan			Triton 6×6mm^2^ Optic Disc Scan			Limit Ratio*	Maestro 12×9mm^2^ Wide Scan			Maestro 6×6mm^2^ Optic Disc Scan			Limit Ratio*
	SD	Limit	CV%	SD	Limit	CV%		SD	Limit	CV%	SD	Limit	CV%	
**Repeatability**														
**Normal Group**														
Average	0.9	2.6	0.9	0.8	2.1	0.7	1.2	1.3	3.7	1.2	0.8	2.2	0.8	1.7
Superior	2.9	8.2	2.3	2.1	6.0	1.6	1.4	3.9	11.0	3.0	2.5	7.1	1.9	1.5
Nasal	1.7	4.8	1.9	1.4	3.9	1.6	1.2	2.0	5.7	2.3	1.5	4.1	1.7	1.4
Inferior	2.2	6.3	1.6	1.4	4.0	1.0	1.6	2.7	7.6	1.9	2.1	5.8	1.5	1.3
Temporal	1.0	2.9	1.4	0.8	2.2	1.1	1.3	1.2	3.3	1.7	1.1	3.0	1.5	1.1
**Glaucoma Group**														
Average	1.1	3.0	1.4	1.0	2.8	1.3	1.1	1.5	4.1	1.9	1.2	3.4	1.6	1.2
Superior	2.3	6.4	2.6	2.6	7.2	2.9	0.9	3.4	9.6	4.0	2.4	6.8	2.8	1.4
Nasal	1.8	5.1	2.9	1.7	4.7	2.7	1.1	3.1	8.7	5.1	2.5	6.9	4.1	1.3
Inferior	2.3	6.5	2.5	1.9	5.2	2.0	1.3	3.0	8.3	3.3	2.7	7.6	3.0	1.1
Temporal	1.1	3.0	1.7	0.9	2.5	1.5	1.2	1.2	3.4	2.0	1.4	3.9	2.4	0.9
**Reproducibility**														
**Normal Group**														
Average	1.1	3.1	1.0	1.0	2.9	1.0	1.1	1.4	3.9	1.3	1.0	2.9	1.0	1.3
Superior	3.1	8.7	2.4	2.4	6.6	1.8	1.3	4.2	11.8	3.2	2.6	7.2	2.0	1.6
Nasal	2.1	6.0	2.4	1.7	4.8	2.0	1.3	2.3	6.4	2.6	1.8	5.0	2.1	1.3
Inferior	2.3	6.5	1.7	1.8	5.0	1.3	1.3	3.1	8.6	2.2	2.4	6.6	1.7	1.3
Temporal	1.4	3.9	2.0	1.1	3.0	1.6	1.3	1.3	3.7	1.8	1.3	3.7	1.9	1.0
**Glaucoma Group**														
Average	1.2	3.4	1.6	1.3	3.5	1.7	1.0	1.7	4.8	2.3	1.3	3.7	1.8	1.3
Superior	2.6	7.2	2.9	2.8	7.9	3.2	0.9	3.6	10.1	4.2	2.6	7.2	3.0	1.4
Nasal	2.2	6.1	3.4	1.9	5.4	3.2	1.1	3.7	10.2	6.0	2.9	8.2	4.9	1.2
Inferior	2.5	7.0	2.7	2.1	5.9	2.3	1.2	3.3	9.1	3.6	2.7	7.6	3.0	1.2
Temporal	1.3	3.6	2.1	1.1	3.0	1.8	1.2	1.3	3.7	2.2	1.5	4.2	2.6	0.9

* Limit Ratio: limit of Wide Scan / limit of Cube scan. Unit of SD and limit is μm. Abbreviations: SD, standard deviation.

## Data Availability

The datasets generated and analyzed during the current study are available from the corresponding author on reasonable request.
